# Nutritional value of some raw materials for guinea pigs (*Cavia porcellus*) feeding

**DOI:** 10.1093/tas/txab019

**Published:** 2021-02-08

**Authors:** Jorge Castro-Bedriñana, Doris Chirinos-Peinado

**Affiliations:** Food and Nutritional Security Research Center, Universidad Nacional del Centro del Perú, Av. Mariscal Castilla N° 3909—El Tambo, Huancayo, Perú

**Keywords:** digestible energy, digestible fiber, digestible protein, metabolizable energy, proximal chemical composition, total digestible nutrients

## Abstract

To formulate economically viable foods and achieve high performance in guinea pig production, it is important to know the nutritional value of the feeds, which requires determining their chemical composition, availability of nutrients, and energy content. Chemical analysis, digestibility tests, and digestible energy (DE) and metabolizable energy (ME) content of 63 feeds were determined using male guinea pigs of 4–5 mo of age. The test feeds were fodder, agricultural residues, agro-industrial and kitchen waste, energy flours, and protein flours of animal and vegetable origin. The result showed wide variability in the chemical composition and energy density of the feeds evaluated. In the case of forages, the main feed source for the guinea pigs, the average contents ± SD of crude protein (CP), crude fiber (CF), organic matter (OM), DE, and ME were 18.06 ± 6.50%, 23.08 ± 7.14%, 89.95 ± 2.62%, 2963.71 ± 442.68, and 2430.24 ± 363.00 kcal/kg; for the agro-industrial and kitchen waste, the values were 11.52 ± 4.72%, 22.80 ± 14.61%, 91.37 ± 4.74%, 3006.31 ± 554.01, and 2465.18 ± 454.29 kcal/kg; for protein feeds, the values were 55.18 ± 22.87%, 5.11 ± 5.72%, 91.18 ± 6.92%, 3681.94 ± 433. 24, and 3019.19 ± 355.26 kcal/kg; for energy feeds, the values were 12.73 ± 3.22%, 5.46 ± 1.96%, 95.33 ± 3.32%, 3705.41 ± 171.78, and 3038.43 ± 140.86 kcal/kg. The ME content is directly associated with CP content (*R*^2^ = 0.19) and OM digestibility (*R*^2^ = 0.56) and inversely with CF (*R*^2^ = 0.40) and ash (*R*^2^ = 0.13) content (*P* < 0.01). The results of this study can be used to design feeding programs for family and commercial guinea pig production for meat.

## INTRODUCTION

The domestic guinea pig (*Cavia porcellus*), native to Peru, Argentina, or Brazil (NRC, 1995), contributes to the food security of many populations in the Andean region and other developing countries (Lammers et al., 2009; Matthiesen et al., 2011; Sánchez-Macías et al, 2015), because of its health properties and high content of protein, B-vitamins, linoleic and linolenic acid, and low content of saturated fat and cholesterol (Quevedo, 2012; Avilés et al., 2014).

The market demands guinea pigs of a standard size and quality (Flores-Mancheno, 2016), and to achieve this objective, diets that precisely cover their nutritional requirements should be used (NRC, 1995). However, it is surprising that very little is published about the nutritional needs and nutritional value of their feed (Guevara et al, 2008) and the existing information considers the guinea pig as a laboratory animal or pet (NRC, 1995; Bindelle et al., 2009) and not as a meat producer; therefore, it is important to research the feed, health, and welfare of these animals (Witkowska et al., 2017).

Thanks to the fact that the guinea pig’s digestive tract has a large cecum that may ferment the fiber in the diet (Richarson, 2000), it can consume a variety of feeds, from kitchen waste, agricultural waste, fresh vegetables, to natural grasses, cultivated alone or combined with concentrated supplements (Niba et al, 2004; Kouakou et al., 2013; Witkowska et al., 2017; Sánchez-Macías et al., 2018), and the rate of weight gain and feed utilization is influenced by the chemical composition and energy content of the diet and the animal’s ability to use it (Castro et al., 2017, 2018); therefore, one of the greatest challenges for a nutritionist is to formulate economically viable diets that adequately satisfy the animal’s nutritional requirements, and it is important to know the nutritional and energy value of the feed and the factors that influence its use.

The proximal chemical composition of feeds is derived from laboratory analyses (Ki et al., 2017), and nutritional information based on in vivo experiments and mathematical estimates (Safwat et al., 2015; INRA, 2017; FEDNA, 2019; National Academy of Sciences, 2019) is available for pigs, birds, and other animal species (Kyntäjä et al., 2014; INRA, 2017; FEDNA, 2019) but not for guinea pigs for meat production.

Therefore, work was conducted to determine the proximal composition, digestibility of nutrients, and contribution of DE and ME of different feeds used in the diets of meat guinea pigs and to evaluate the association between the content of crude protein (CP), crude fiber (CF), ash, and the digestibility of organic material with ME content.

## MATERIALS AND METHODS

### Animal Handling

All digestibility tests met ethical standards for animal research. The biological evaluation room was well ventilated and illuminated; the handling and treatment of experimental animals followed ethical standards of animal welfare and care in research. After the study, all of the guinea pigs returned to the farm.

The research was approved by the Specialized Research Institute of the Faculty of Animal Science of the Universidad Nacional del Centro del Perú. It is part of the project “Bioenergetic Valuation of Inputs for the Feeding of Guinea pigs”, Research Line in Sustainable Animal Production. Project Code N° 1111.

#### Place and period of the study.

A total of 63 digestibility experiments and their corresponding chemical analyses were carried out in the guinea pig program of the Yauris Agricultural Farm and animal nutrition laboratory of the Faculty of Zootechnics of the National University of Central Peru. The university is located in the district and province of Huancayo, at 3240 m of altitude, latitude South 11°51’00’’, longitude West 77°22’24’’, with an average temperature of 11.9 °C, 625 mm/year of rainfall, and 88.2% of relative humidity.

#### Feed inputs.

Sixty-three guinea pig feeds were evaluated, including dry and fresh forages, agro-industrial and kitchen waste, and energetic cereal grain flours and animal and vegetable protein flours.

#### Digestibility tests and calculation of total digestible nutrients.

Experiments were conducted with guinea pigs housed individually in metabolic crates equipped with an individual feeder and a nipple drinker (Castro et al., 2017, 2018). The ingredients that were used as unique dietary components, in “in vivo” direct digestibility tests (Safwat et al., 2015; Fan et al., 2017), were fodder and agricultural and kitchen waste. Protein and energy meals were included in 10% of a reference ration based on barley flour and their digestibility coefficients were determined by the difference method, considering that the indigestibility of barley flour is the same when combined with the experimental feed (Fan et al., 2017).

The digestibility coefficients were determined using the method of total excrement collection (Stein et al., 2007; Castro et al., 2017, 2018), which considers a pre-experimental phase of 7 d, gradually substituting the *Lolium multiflorum* that the guinea pigs had been consuming with the feed in the study plus drinking water with vitamin C (Richardson, 2000; Bindele et al., 2009; Balsiger et al., 2016; Frikke-Schmidth et al., 2016). During the experimental phase, accurate measurement of feed intake and collection of fecal material was performed daily for 7 d and feces were weighed and stored at −18 °C for subsequent chemical analysis (Bindele et al., 2009).

Total digestible nutrients (TDN) are calculated as the sum of the products of the organic components of the proximal analysis [CP, ether extract (EE), CF, and the nitrogen-free extract (NFE)] multiplied by their digestibility coefficients. The product of the multiplication of EE by its digestibility is multiplied by the factor 2.25, which is the times of energy released by fats compared to proteins and carbohydrates. The partial results are divided by 100 to express the TDN as a percentage (Castro et al., 2017, 2018).

#### Proximal chemical analysis and estimation of digestible and metabolizable energy.

Dry matter (DM), CP, EE, CF, NFE, and ash contents in feed and feces samples were determined in accordance with the standard methods of the Association of Official Analytical Chemists procedures (AOAC, 2005). Moisture content was determined gravimetrically by drying the samples in an oven at 100 °C to a constant weight. The dried samples were subjected to other chemical analyses. CP content (*N* × 6.25) was determined in accordance with the Kjeldahl method (method no. 978.04), EE was determined in accordance with the Soxhlet extract method using petroleum ether as the extract agent (60–80 °C; method no. 930.09), CF was determined using H_2_SO_4_ and NaOH digestion (method no. 978.10), and ash content was assayed by incinerating the samples in a muffle furnace at 550 °C (method no. 930.05). Organic matter (OM) was computed as 100 minus the content of ash and water (Fan et al., 2017). Digestible energy (DE) was estimated by multiplying the percentage of TDN by 44.09 (Weiss and Tebbe, 2019) and metabolizable energy (ME) by multiplying the DE content by 0.82 (Hales, 2019), both values were expressed in kcal/kg.

### Statistical Analysis

The calculations of digestible nutrients and energy assessment of feed were made in Excel for Windows. Trend lines and determination coefficients (*R*^2^) between ME content and CF, CP, ash, and OM digestibility contents were determined. One-way ANOVA was used for analysis between the digestibility coefficients of DM, CP, EE, NFE, and OM, and Tukey significance tests with a 5% probability level in SPSS 23.

## RESULTS

### Proximal Composition and Digestibility Coefficients of Guinea Pig Feed

The contents of DM, CP, EE, NFE, CF, and ash vary according to the type of feed evaluated (dry fodder, green fodder, protein feed, and energy feed or kitchen and agro-industrial waste). The OM content of the 63 feeds evaluated ranged from 72.90% to 98.00% (Tables 1 and 2).

**Table 1.** Proximal chemical composition and digestibility coefficients of guinea pig food (%): dried and green forages

Food	DM	CP	EE	CF	NFE	A	OM	DMD	CPD	EED	CFD	NFED	OMD
Dried fodder													
Flat corn: leaves (*Zea mays* L.)	80.83	13.96	3.97	34.32	37.93	9.82	90.18	54.66	65.37	77.18	55.46	53.98	52.16
Flat corn: Stem	76.44	7.04	1.11	22.73	62.41	6.71	93.29	62.20	57.54	72.33	59.73	66.87	54.76
Flat corn	79.66	9.36	2.57	28.99	51.96	7.12	92.88	57.38	64.79	78.41	57.91	64.99	54.04
Alfalfa hay (*Medicago sativa*)	88.36	16.79	2.25	30.26	40.73	9.97	90.03	59.00	22.40	40.70	78.90	56.80	53.78
Corn: dried pancake	87.03	5.93	1.78	34.21	51.41	6.67	93.33	58.36	57.64	66.43	56.10	45.30	56.36
Maca stubble (*Lepidium meyenii*)	88.70	6.97	3.58	36.35	44.52	8.58	91.42	74.79	68.00	75.27	71.78	77.08	74.21
Green forages													
*Phalaris tuberoarundinacea*: aerial part	18.40	23.75	4.38	26.60	35.17	10.10	89.90	50.07	74.37	56.64	49.84	58.91	60.20
*Phalaris tuberoarundinacea*: leaves	26.10	29.03	5.80	24.97	28.90	11.30	88.70	73.26	83.77	67.18	68.67	69.40	73.90
*Phalaris tuberoarundinacea*: stem	14.90	16.06	1.81	34.21	35.62	12.30	87.70	52.12	59.28	20.15	49.63	51.83	51.70
Smooth cabbage (*Brassica oleracea* var. capitata)	8.39	18.68	1.23	12.62	61.45	6.02	93.98	85.67	84.06	19.97	52.85	95.46	86.50
Smooth cabbage (in Creole guinea pigs)	8.39	18.68	1.23	12.62	61.45	6.02	93.98	82.35	68.95	32.59	64.08	93.46	83.50
Curly cabbage (*Brassica oleracea* var. sbauda)	18.10	13.09	3.43	10.65	60.83	12.00	88.00	90.70	83.05	51.66	90.38	94.22	91.80
Carrot leaves (*Daucus carota*)	20.93	15.71	3.78	14.25	47.96	18.30	81.70	90.21	86.49	88.76	81.91	93.77	90.10
Root carrot	11.90	14.09	0.60	17.51	58.88	8.92	91.08	97.93	96.05	87.97	97.98	98.78	98.10
Cattail: without inflorescence (*Scirpus californicus*)	30.69	14.68	2.17	27.89	43.86	11.40	88.60	76.67	83.69	79.31	68.16	78.53	78.20
Cattail: with inflorescence	31.53	11.91	1.73	30.85	43.01	12.50	87.50	64.62	77.05	62.53	54.34	65.73	63.70
Preflowering cattails (2% NaOH)	30.87	13.87	2.11	28.15	44.29	11.58	88.42	65.27	75.06	64.32	59.37	66.70	66.35
Preflowering cattails (3% NaOH)	30.87	13.87	2.11	28.15	44.29	11.58	88.42	69.88	78.46	65.02	60.68	68.89	71.24
Preflowering cattails (4% NaOH)	30.87	13.87	2.11	28.15	44.29	11.58	88.42	70.86	81.25	67.56	64.21	69.93	71.96
Green alfalfa	21.50	25.87	2.92	22.16	38.49	10.56	89.44	60.59	74.76	48.46	31.04	78.01	61.83
Italian rye grass (*Lolium multiflorum*)	18.46	22.16	3.56	17.94	44.20	12.14	87.86	69.78	80.71	57.31	57.64	78.85	71.95
English rye gras (*Lolium perenne*)	13.54	25.38	2.86	20.64	40.58	10.54	89.46	70.90	84.22	58.93	60.88	82.33	72.92
English rye gras (guinea pigs of 1 month)	13.54	25.38	2.86	20.64	40.58	10.54	89.46	76.01	88.22	54.63	53.99	84.28	75.75
English rye gras (guinea pigs of 2 month)	13.54	25.38	2.86	20.64	40.58	10.54	89.46	72.59	80.66	50.99	53.01	79.94	73.01
English rye gras (guinea pigs of 3 month)	13.54	25.38	2.86	20.64	40.58	10.54	89.46	74.86	80.82	54.45	55.71	82.27	74.84
English rye gras (guinea pigs of 4 month)	13.54	25.38	2.86	20.64	40.58	10.54	89.46	77.16	83.45	56.45	60.70	83.77	77.48
English rye gras (guinea pigs of 5 month)	13.54	25.38	2.86	20.64	40.58	10.54	89.46	77.56	83.69	59.39	61.28	82.71	77.29
English rye gras (guinea pigs of 6 month)	13.54	25.38	2.86	20.64	40.58	10.54	89.46	77.70	83.20	59.25	61.95	83.17	77.49
White clover (*Trifolium repens*) Pre-flower	17.48	19.90	3.12	14.15	55.27	7.56	92.44	70.23	73.18	54.68	48.79	87.13	73.03
White clover (*Trifolium repens*) Start-flower	20.51	21.73	2.96	17.20	47.54	10.57	89.43	68.22	70.82	40.15	39.13	95.84	69.44
Hydroponic barley forage (*Hordeum vulgare*)	17.32	15.12	3.52	16.08	60.92	4.36	95.64	74.15	78.21	63.39	75.89	75.57	76.12

All values are shown in percentage.

A, ash; DMD, DM digestibility coefficient; CPD, CP digestibility coefficient; EED, EE digestibility coefficient; CFD, CF digestibility coefficient; NFED, NFE digestibility coefficient; OMD, OM digestibility coefficient.

**Table 2.** Proximal chemical composition and digestibility coefficients of guinea pig food (%): protein and energy foods, agroindustrial and kitchen waste

Food	DM	CP	EE	CF	NFE	A	OM	DMD	CPD	EED	CFD	NFED	OMD
Protein foods^*a*^													
Beef blood meal: raw	88.12	95.16	0.68	0.00	0.00	4.16	95.84	92.58	83.52	93.93			92.80
Beef blood meal: coocked	88.15	92.25	0.56	0.00	0.00	7.19	92.81	87.68	78.18	94.36			81.90
Donkey blood meal: raw	91.55	78.78	2.63	0.00	14.59	4.00	96.00	100.00	99.06	99.58		100.00	100.00
Donkey blood meal: coocked	91.75	85.23	2.00	0.00	8.89	3.88	96.12	95.83	87.28	96.51		99.47	91.30
Fish viscera meal: raw	91.70	40.23	25.84	0.00	0.00	22.50	77.50	71.40	73.94	82.62			67.10
Fish viscera meal: coocked	91.23	74.12	7.97	0.00	0.00	17.91	82.09	87.66	100.00	81.00			84.12
Fish meal 1st.	89.40	52.04	13.61	0.00	7.25	27.10	72.90	64.23	77.31	81.77			66.90
Fish meal 3rd.	77.16	66.90	10.00	0.00	14.10	9.00	91.00	64.14	91.87	69.67		33.24	69.26
Earthworm meal (10%) (*Eisenia foetida*)	77.16	66.90	10.00	0.00	14.10	9.00	91.00	71.88	94.05	75.12		49.43	74.09
Earthworm meal (20%)	97.22	38.75	21.40	9.10	26.15	4.60	95.40	69.03	88.69	79.86	35.26	47.60	69.56
Tarwi flour not embittered (*Lupinus mutabilis*)	97.22	38.75	21.40	9.10	26.15	4.60	95.40	77.79	93.49	82.96	53.57	61.25	78.49
Tarwi flour not embittered + Met (0.15%)	97.22	38.75	21.40	9.10	26.15	4.60	95.40	69.75	89.33	80.93	36.97	48.92	71.38
Tarwi flour not embittered + Lis (0.15%)	97.22	38.75	21.40	9.10	26.15	4.60	95.40	78.14	93.40	84.39	47.49	63.82	78.89
Tarwi flour not embittered with Met + Lis	96.64	32.87	20.60	12.60	27.23	6.70	93.30	53.95	73.58	74.80	40.99	27.40	55.97
Tarwi flour without not embittered	96.64	32.87	20.60	12.60	27.23	6.70	93.30	60.22	78.56	79.87	47.70	35.36	62.08
Tarwi flour without not embittered + Met	96.64	32.87	20.60	12.60	27.23	6.70	93.30	55.12	74.48	69.76	42.55	31.65	56.62
Tarwi flour without not embittered + Lis	96.64	32.87	20.60	12.60	27.23	6.70	93.30	67.36	88.81	82.57	58.10	38.09	68.49
Energetic foods^*a*^													
Grain barley (*Hordeum vulgare*)	91.70	16.31	2.08	7.01	70.98	3.62	96.38	79.06	63.72	65.99	53.75	88.10	80.30
Barley flour	88.63	11.81	3.50	6.10	76.59	2.00	98.00	83.75	62.56	71.81	77.26	87.69	84.92
Yellow Maize (*Zea mays* var. Indurata)	88.25	10.07	4.54	3.26	73.75	8.38	91.62	92.21	87.60	74.35	53.47	93.92	83.36
Kitchen and agro-industrial waste													
Potato peel (*Solanum tuberosum*) improved guinea pigs	26.70	10.58	0.67	9.73	70.58	8.44	91.56	83.27	55.61	22.21	61.82	92.34	84.30
Potato peel (Creole guinea pigs)	26.70	10.58	0.67	9.73	70.58	8.44	91.56	89.00	48.84	50.75	82.29	96.87	89.40
Carrot peel (improved guinea pigs)	10.71	5.08	1.62	10.48	76.75	6.07	93.93	77.55	24.48	41.66	41.21	89.82	80.00
Carrot peel (creole guinea pigs)	10.71	5.08	1.62	10.48	76.75	6.07	93.93	88.43	52.00	49.72	54.11	97.17	89.10
Dried fava bean peel (*Vicia faba*)	88.93	6.22	0.45	39.57	50.73	3.03	96.97	72.18	12.38	78.16	83.80	66.27	75.70
Dry pea peel (*Pisum sativum*)	89.85	7.06	1.39	42.03	46.01	3.51	96.49	76.06	40.57	77.67	74.72	70.75	78.40
Kiwicha peel (*Amaranthus caudatus*)	89.95	16.47	2.34	22.74	43.15	15.30	84.70	51.11	64.27	37.42	42.02	49.56	50.80
Quinoa husk (*Chenopodium quinoa*)	90.07	14.27	2.69	16.63	50.41	16.00	84.00	52.24	54.62	54.07	31.53	58.78	51.50
Tarwi husk (*Lupinus mutabilis*)	91.02	17.52	4.93	37.88	23.27	16.40	83.60	81.94	65.55	55.73	85.79	85.57	82.60
Morón bran (from roasted and split barley)	90.15	13.86	3.66	47.36	28.69	6.43	93.57	70.92	56.71	60.00	79.24	59.81	73.80
Wheat bran (*Triticum aestivum*)	89.48	14.56	4.22	15.69	56.77	8.76	91.24	71.54	78.10	33.20	60.10	72.80	71.88
Wheat byproduct (*Triticum aestivum*)	89.04	16.93	4.13	11.22	62.61	5.11	94.89	68.42	70.32	64.89	30.43	74.38	70.56

All values are shown in percentage.

A, ash; DMD, DM digestibility coefficient; CPD, CP digestibility coefficient; EED, EE digestibility coefficient; CFD, CF digestibility coefficient; NFED, NFE digestibility coefficient; OMD, OM digestibility coefficient.

^*a*^Foods evaluated in digestibility tests by the difference method. They were included in 10% in the reference diets (90% barley flour + 10% study feed).

The digestibility coefficients determined in this study show that the guinea pigs have a wide capacity to use diverse types of feed, from high fiber feeds, such as moron bran (CF 47.36%) and dry pea shells (CF 42.03%), to high protein feeds, such as cattle blood meal (CP 95.16%) or donkey meal (CP 85.23%), that can be used as the main protein sources in guinea pig diets. Our results indicate that NFE is composed of noncomplex carbohydrates and is more digestible than raw fiber (*P* > 0.05); the average digestibility of NFE was 72.15%, while that of CF was 58.82%.

**Figure 1.**
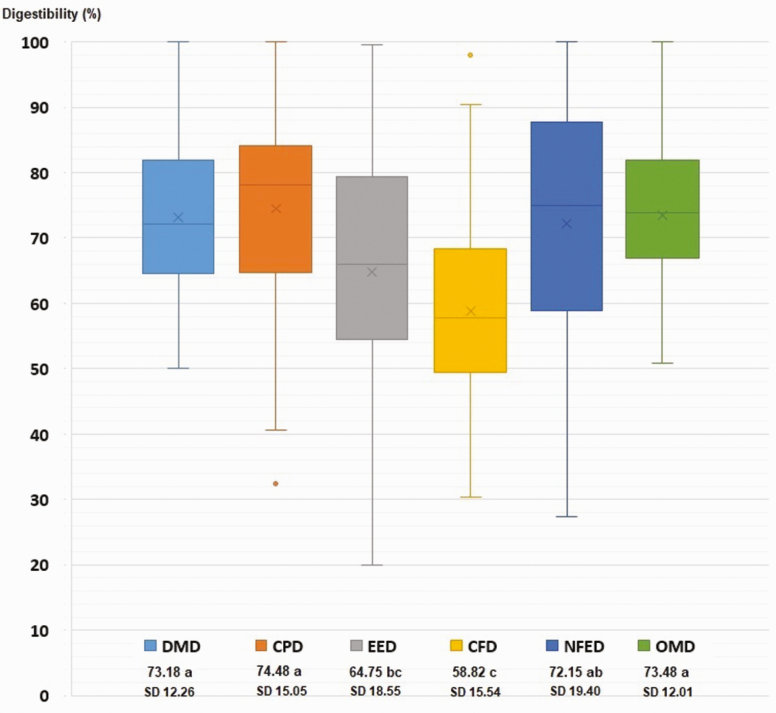
Diagram of boxes and whiskers for the distribution of digestibility coefficients of DM, OM, and proximal organic components of guinea pig food. Averages and SD are shown. a, b, c, Average values with different letters vary statistically (*P* < 0.05).

Figure 1 shows that the average digestibility coefficients ± SD of the DM, CP, EE, CF, NFE, and OM of study feeds registered highly significant differences (*P* < 0.05), with the digestibilities of the CP, DM, and OM being similar and greater than the digestibility registered for the fibrous fraction of the feeds.

#### Proximal digestible components and energy content of guinea pig feed.

**Figure 2.**
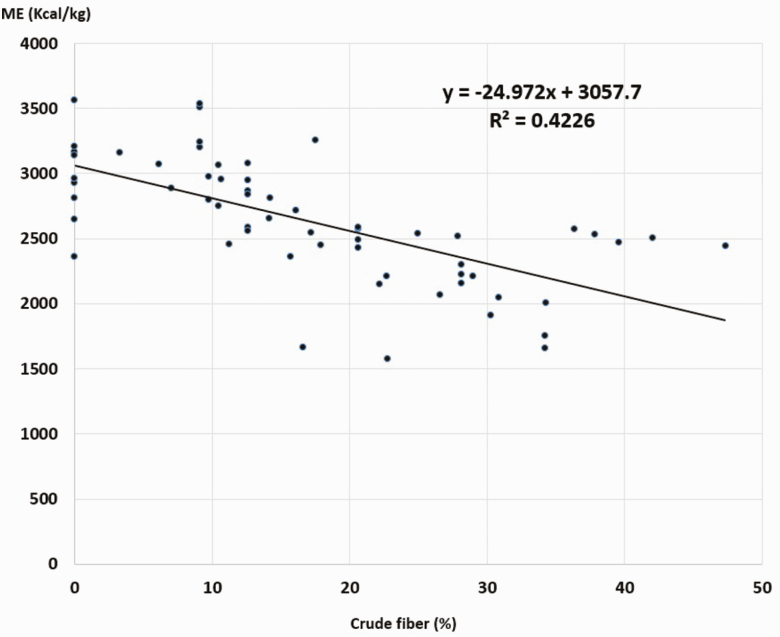
Association between ME and CF contents in guinea pig foods. *R* = 0.650. ANOVA regression *P* < 0.05).

**Figure 3.**
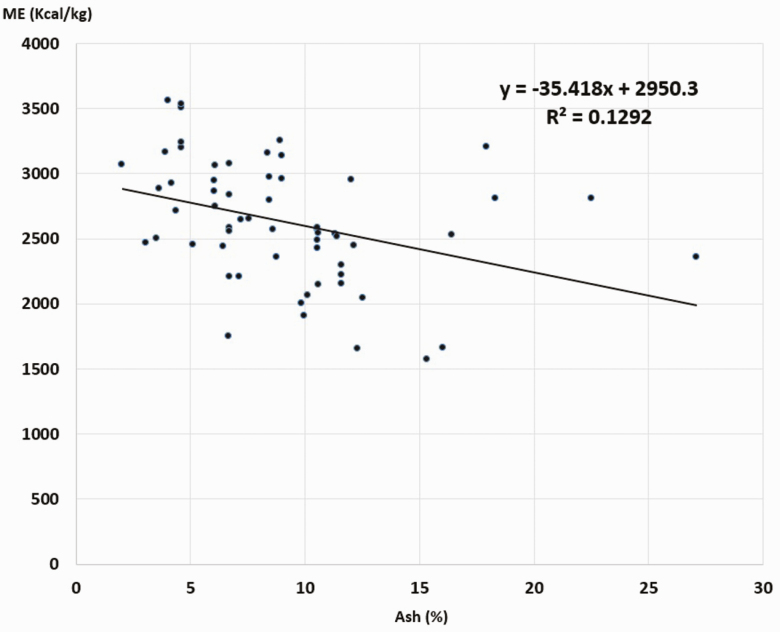
Association between ME and ash contents in guinea pig foods. *R* = 0.359. ANOVA regression *P* < 0.05.

**Figure 4.**
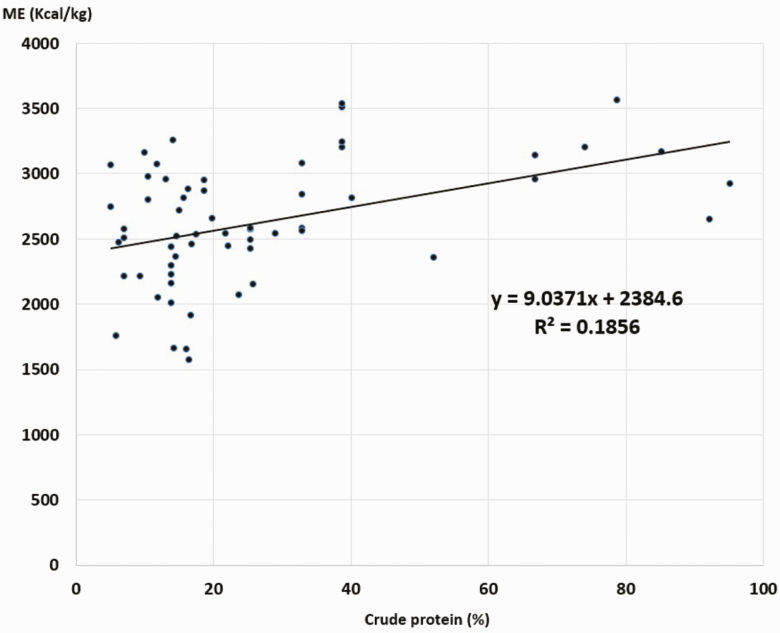
Association between ME and CP contents in guinea pig foods. *R* = 0.431. ANOVA regression *P* < 0.05.

**Figure 5.**
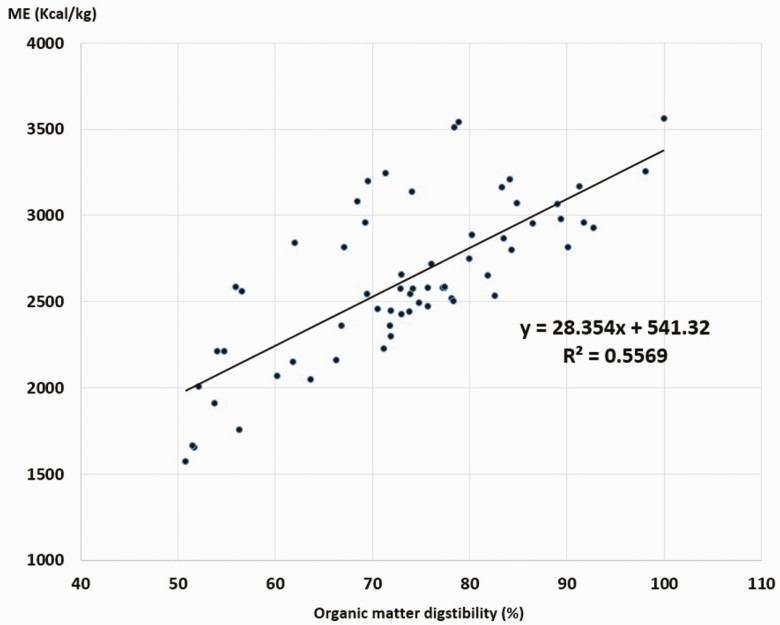
Association between ME content and OM digestibility in guinea pig foods. *R* = 0.746. ANOVA regression *P* < 0.05.

The guinea pigs make good use of all types of feed, with high digestibility of DM, CP, and OM (Tables 3 and 4). The ME contents of the 63 feeds evaluated were between 1572.54 and 3561.95 kcal/kg, with an average ± SD = 2628.01 ± 453.62 kcal/kg. Figures 2–5 show the inverse relationship between ME intake and CF and A contents (*P* < 0.05) and a direct association with CP contents and OM digestibility (*P* < 0.05).

**Table 3.** Digestible components and energy content of guinea pig food: dried and green forages

Food	DCP	DEE	DCF	DNFE	DOM	GE (kcal/kg)	TDN %	DE (kcal/kg)	ME (kcal/kg)
Dried fodder									
Flat corn: leaves (*Zea mays* L.)	9.13	3.06	19.03	20.47	47.04	4127.18	55.53	2448.24	2007.56
Flat corn: stem	4.05	0.80	13.58	41.73	51.09	3995.25	61.17	2696.87	2211.44
Flat corn	6.06	2.02	16.79	33.77	50.19	4091.48	61.16	2696.34	2211.00
Alfalfa hay (*Medicago sativa*)	3.76	0.92	23.88	23.13	48.42	4076.87	52.83	2329.33	1910.05
Corn: dried pancake	3.42	1.18	19.19	23.29	52.60	4013.97	48.56	2140.97	1755.60
Maca stubble (*Lepidium meyenii*)	4.74	2.69	26.09	34.32	67.84	4045.90	71.21	3139.68	2574.54
Green forages									
*Phalaris tuberoarundinacea*: aerial part	17.66	2.48	13.26	20.72	54.12	4298.04	57.22	2522.87	2068.75
*Phalaris tuberoarundinacea*: leaves	24.32	3.90	17.15	20.06	65.55	4408.58	70.29	3099.04	2541.21
*Phalaris tuberoarundinacea*: stem	9.52	0.36	16.98	18.46	45.34	3948.59	45.78	2018.50	1655.17
Smooth cabbage (*Brassica oleracea* var. capitata)	15.70	0.25	6.67	58.66	81.29	4217.25	81.58	3597.08	2949.60
Smooth cabbage (in Creole guinea pigs)	12.88	0.40	8.09	57.43	78.47	4217.25	79.30	3496.33	2866.99
Curly cabbage (*Brassica oleracea* var.sbauda)	10.87	1.77	9.63	57.31	80.78	3999.23	81.80	3606.46	2957.29
Carrot leaves (*Daucus carota*)	13.59	3.36	11.67	44.97	73.61	3801.40	77.78	3429.36	2812.07
Root carrot	13.53	0.53	17.16	58.16	89.35	3991.52	90.04	3969.82	3255.25
Cattail: without inflorescence (*Scirpus californicus*)	12.29	1.72	19.01	34.44	69.29	3980.32	69.61	3069.15	2516.71
Cattail: with inflorescence	9.18	1.08	16.76	28.27	55.74	3868.02	56.64	2497.48	2047.93
Preflowering cattails (2% NaOH)	10.41	1.36	16.71	29.54	58.67	3956.86	59.72	2632.99	2159.05
Preflowering cattails (3% NaOH)	10.88	1.37	17.08	30.51	62.99	3956.86	61.56	2714.27	2225.70
Preflowering cattails (4% NaOH)	11.27	1.43	18.08	30.97	63.63	3956.86	63.52	2800.77	2296.63
Green alfalfa	19.34	1.42	6.88	30.03	55.30	4232.80	59.43	2620.21	2148.58
Italian rye grass (*Lolium multiflorum*)	17.89	2.04	10.34	34.85	63.22	4141.94	67.67	2983.49	2446.46
English rye gras (*Lolium perenne*)	21.38	1.69	12.57	33.41	65.23	4222.66	71.14	3136.67	2572.07
English rye gras (guinea pigs of 1 month)	22.39	1.56	11.14	34.20	67.77	4222.66	71.25	3141.41	2575.96
English rye gras (guinea pigs of 2 month)	20.47	1.46	10.94	32.44	65.31	4222.66	67.13	2959.92	2427.14
English rye gras (guinea pigs of 3 month)	20.51	1.56	11.50	33.39	66.95	4222.66	68.90	3037.79	2490.99
English rye gras (guinea pigs of 4 month)	21.18	1.61	12.53	33.99	69.31	4222.66	71.33	3145.14	2579.01
English rye gras (guinea pigs of 5 month)	21.24	1.70	12.65	33.56	69.14	4222.66	71.27	3142.48	2576.83
English rye gras (guinea pigs of 6 month)	21.12	1.69	12.79	33.75	69.32	4222.66	71.47	3150.93	2583.76
White clover (*Trifolium repens*) preflower	14.56	1.71	6.90	48.16	67.51	4270.68	73.46	3238.93	2655.93
White clover (*Trifolium repens*) start-flower	15.39	1.19	6.73	45.56	62.10	4168.23	70.36	3101.99	2543.63
Hydroponic barley forage (*Hordeum vulgare*)	11.83	2.23	12.20	46.04	72.80	4346.20	75.09	3310.55	2714.65

All values are shown in percentage.

DCP, digestible CP; DEE, digestible ethereal extract; DCF, digestible CF; DNFE, digestible NFE; DOM, digestible OM. GE, gross energy (kcal/kg DM).

**Table 4.** Digestible components and energy content of guinea pig food: protein and energy foods, agro-industrial and kitchen waste

Food	DCP	DEE	DCF	DNFE	DOM	GE (kcal/kg)	TDN %	DE (kcal/kg)	ME (kcal/kg)
Protein foods^*a*^									
Beef blood meal: raw	79.48	0.64	0.00	0.00	88.94	5487.36	80.91	3567.53	2925.38
Beef blood meal: coocked	72.12	0.53	0.00	0.00	76.01	5310.33	73.31	3232.24	2650.43
Donkey blood meal: raw	78.04	2.62	0.00	14.59	96.00	5333.24	98.52	4343.84	3561.95
Donkey blood meal: coocked	74.39	1.93	0.00	8.84	87.76	5408.60	87.57	3861.16	3166.15
Fish viscera meal: raw	29.75	21.35	0.00	0.00	52.00	4696.23	77.78	3429.38	2812.09
Fish viscera meal: coocked	74.12	6.46	0.00	0.00	69.05	4966.05	88.65	3908.37	3204.87
Fish meal 1st.	40.23	11.13	0.00	0.00	48.77	4529.26	65.27	2877.85	2359.84
Fish meal 3rd.	61.46	6.97	0.00	4.69	63.03	5321.40	81.82	3607.60	2958.23
Earthworm meal (10%) (*Eisenia foetida*)	62.92	7.51	0.00	6.97	67.42	5321.40	86.79	3826.62	3137.83
Earthworm meal (20%)	34.37	17.09	3.21	12.45	66.36	5644.20	88.48	3900.91	3198.74
Tarwi flour not embittered (*Lupinus mutabilis*)	36.23	17.75	4.87	16.02	74.88	5644.20	97.06	4279.57	3509.25
Tarwi flour not embittered + Met (0.15%)	34.62	17.32	3.36	12.79	68.10	5644.20	89.74	3956.64	3244.44
Tarwi flour not embittered + Lis (0.15%)	36.19	18.06	4.32	16.69	75.26	5644.20	97.84	4313.62	3537.17
Tarwi flour not embittered with Met + Lis	24.19	15.41	5.16	7.46	52.22	5422.42	71.48	3151.61	2584.32
Tarwi flour without not embittered	25.82	16.45	6.01	9.63	57.92	5422.42	78.48	3460.23	2837.39
Tarwi flour without not embittered + Met	24.48	14.37	5.36	8.62	52.83	5422.42	70.79	3121.35	2559.51
Tarwi flour without not embittered + Lis	29.19	17.01	7.32	10.37	63.90	5422.42	85.16	3754.51	3078.70
Energetic foods^*a*^									
Grain barley (*Hordeum vulgare*)	10.39	1.37	3.77	62.53	77.39	4320.70	79.78	3517.60	2884.43
Barley flour	7.39	2.51	4.71	67.16	83.22	4388.96	84.92	3744.03	3070.11
Yellow Maize (*Zea mays* var. Indurata)	8.82	3.38	1.74	69.27	76.37	4153.62	87.43	3854.58	3160.76
Kitchen and agro-industrial waste									
Potato peel (*Solanum tuberosum*) in improved guinea pigs	5.88	0.15	6.02	65.17	77.19	3958.08	77.41	3412.88	2798.56
Potato peel (Creole guinea pigs)	5.17	0.34	8.01	68.37	81.85	3958.08	82.31	3629.05	2975.82
Carrot peel (improved guinea pigs)	1.24	0.67	4.32	68.94	75.14	4016.65	76.02	3351.62	2748.33
Carrot peel (creole guinea pigs)	2.64	0.81	5.67	74.58	83.69	4016.65	84.70	3734.54	3062.32
Dried fava bean peel (*Vicia faba*)	0.77	0.35	33.16	33.62	73.41	4098.69	68.34	3013.10	2470.74
Dry pea peel (*Pisum sativum*)	2.86	1.08	31.40	32.55	75.65	4141.33	69.25	3053.24	2503.66
Kiwicha peel (*Amaranthus caudatus*)	10.59	0.88	9.56	21.39	43.03	3857.90	43.50	1917.74	1572.54
Quinoa husk (*Chenopodium quinoa*)	7.79	1.45	5.24	29.63	43.26	3812.20	45.94	2025.55	1660.95
Tarwi husk (*Lupinus mutabilis*)	11.48	2.75	32.50	19.91	69.05	3964.28	70.08	3089.63	2533.50
Morón bran (from roasted and split barley)	7.86	2.20	37.53	17.16	69.05	4248.45	67.49	2975.57	2439.97
Wheat bran (*Triticum aestivum*)	11.37	1.40	9.43	41.33	65.58	4193.24	65.28	2878.28	2360.19
Wheat byproduct (*Triticum aestivum*)	11.91	2.68	3.41	46.57	66.95	4376.13	67.92	2994.53	2455.52

All values are shown in percentage.

DCP, digestible CP; DEE, digestible ethereal extract; DCF, digestible CF; DNFE, digestible NFE; DOM, digestible OM; GE, gross energy (kcal/kg DM).

^*a*^Foods evaluated in digestibility tests by the difference method. They were included in 10% in the reference diets (90% barley flour + 10% study feed).

## DISCUSSION

The food intake and productive yield of the guinea pigs depend on the nutritional value and energy density of the diet. The feed represents about 70% of the cost of guinea pig fattening, and it is important to know the chemical composition and energy contribution of the feed in correspondence to the requirements of the guinea pigs (NRC, 1995).

In the current study, the nutritional value of the feed, determined through proximal analysis and digestibility tests, has allowed results to be obtained for use in guinea pig diet formulation (Castro et al., 2017, 2018; Witkowska et al, 2017; Sánchez-Macías et al., 2018), as reported in other animal species (Fortes et al., 2010; Safwat et al., 2015; Stergiadis et al., 2015; Fan et al., 2017). The DM content of feeds, their proximal composition, and digestibility varies according to different factors, such as the species of grass, plant parts, cutting age, type, and origin of the feed, whether it is of animal or plant origin (Gadberry, 2015).

Even when the forages are characterized by their high fiber content, generally greater than that of CP, the guinea pig, because it is a postgastric fermenter, has a functional cecum that contains between 40% and 65% of the intake and, thanks to the fermentative activity of a wide variety of bacteria, fungi, and protozoa, the guinea pig can partly hydrolyze and ferment cellulose and other fiber components (Snipes, 1982; Richardson 2000; Vella, 2012; Witkowska et al, 2017). If there is not enough fiber in the diet, the teeth do not wear out properly and can cause malocclusions that make it impossible to eat; therefore, forages should constitute the majority of the diet and be provided on an ad libitum basis (Meredith and Johnson-Delaney, 2010).

The values of CP do not provide information about the real content of protein and nonprotein in the feed, so the digestible protein can be used in the formulation of rations for the guinea pigs. In this study, the average CP digestibility was 73.53, which was also greater than the CF digestibility of 58.82% (*P* > 0.05).

TDN were determined based on the results of digestibility tests, which are still used as a standard system to express the energy value of feeds for many species (NRC, 1995, 2012); however, DE and ME values are the most recommended for guinea pig feeding (NRC, 1995).

This study shows that guinea pigs use pasture and forage, agro-industrial waste, and kitchen waste well, improving their nutritional contribution when concentrates with protein sources of animal origin are included (Fortes et al., 2010) or when vegetable supplements are complemented with essential amino acids, as is the case with lupine flour, or when fibrous feeds are treated with NaOH (Tables 3 and 4).

In this study, it was observed that feeds rich in fiber decrease the digestibility of the other components and the content of ME, results observed in several reports (Fortes et al., 2010). The progress of the phenological state of the plant increases the content of the cell walls to the detriment of the nitrogen cell content (Pérez, 2013; Beltrán et al., 2018). It has also been observed that the proportion of protein, EE, fiber, and ash was higher in the leaves than in the stems, as in the case of *Phalaris tuberoarundinacea* and flat corn (*Zea mays*).

Results also demonstrated that the higher the CF and cellulose concentration in the ingredient, the lower was the ME, and when feeds had greater CP concentration and OM digestibility, the ME contribution increases significantly (*P* < 0.05). Similar results have been observed in other monogastric animals (Castrillo et al., 2001; Agyekum and Nyachoti, 2017; Ginindza et al., 2017).

## CONCLUSIONS

The feed of the guinea pigs contains very variable amounts of protein, fiber, fat, and ME, and the majority is of a fibrous nature. In general, feeds high in fiber have a lower nutritional value and affect the utilization of ME. However, the guinea pigs ferment the fiber better than other monogastric animals because they have a functional cecum. Feeds rich in fiber and ash are associated with less utilization of nutrients and ME, and feeds rich in raw protein are associated with greater utilization of energy.

## LIMITATIONS OF THE STUDY

Feeds present variations in their composition due to the effects of climate, geochemistry, tillage system, fertilization, cutting season, variety, handling, and preparation, among others; consequently, an estimate of the nutritional value of feeds is approximate but reliable and can be used to formulate rations. In any case, feeding tests can be done periodically to measure the ED and ME of the feed and guarantee the accuracy in the formulation of rations.

## ANIMAL WELFARE STATEMENT

The authors confirm that the journal’s ethical policies have been followed as indicated on the journal’s author guidelines page.
